# Weighting the structural connectome: Exploring its impact on network properties and predicting cognitive performance in the human brain

**DOI:** 10.1162/netn_a_00342

**Published:** 2024-04-01

**Authors:** Hila Gast, Yaniv Assaf

**Affiliations:** Sagol School of Neuroscience, Tel Aviv University, Tel Aviv, Israel; The Strauss Center for Neuroimaging, Tel Aviv University, Tel Aviv, Israel; School of Neurobiology, Biochemistry and Biophysics, Faculty of Life Sciences, Tel Aviv University, Tel Aviv, Israel

**Keywords:** Structural connectome, Network properties, Cognitive performances, HCP, Prediction model, Axon diameter distribution

## Abstract

Brain function does not emerge from isolated activity, but rather from the interactions and exchanges between neural elements that form a network known as the connectome. The human connectome consists of structural and functional aspects. The structural connectome (SC) represents the anatomical connections, and the functional connectome represents the resulting dynamics that emerge from this arrangement of structures. As there are different ways of weighting these connections, it is important to consider how such different approaches impact study conclusions. Here, we propose that different weighted connectomes result in varied network properties, and while neither superior the other, selection might affect interpretation and conclusions in different study cases. We present three different weighting models, namely, number of streamlines (NOS), fractional anisotropy (FA), and axon diameter distribution (ADD), to demonstrate these differences. The later, is extracted using recently published AxSI method and is first compared to commonly used weighting methods. Moreover, we explore the functional relevance of each weighted SC, using the Human Connectome Project (HCP) database. By analyzing intelligence-related data, we develop a predictive model for cognitive performance based on graph properties and the National Institutes of Health (NIH) toolbox. Results demonstrate that the ADD SC, combined with a functional subnetwork model, outperforms other models in estimating cognitive performance.

## Author Summary

This study compares different approaches of weighting connections in the human brain structural connectome. The three chosen methods for weighting are based on diffusion MRI data and include the number of streamlines, FA index, and axon diameter distribution, which is measured using the recently published AxSI method. We examine local and global properties of the different resulted weighted graphs and discuss how they varied from each other. Lastly, we use each of the weighting connectomes to predict cognitive performance by using the Human Connectome Project (HCP) data set.

## INTRODUCTION

The human brain can be conceptualized as a network of cells or brain areas that are interconnected through axons, fiber tracts, or temporally correlated activation (Fornito et al., 2016; Sporns et al., 2005). This perspective, known as the connectome, considers the brain not just as a collection of elements but also in terms of their interactions and connections (Sporns et al., 2004, 2005). Since the introduction of the connectome as a field of study in neuroscience, researchers worldwide have sought to characterize it and establish the most appropriate way of defining it. Understanding the underlying architecture of such a network is a crucial issue in neuroscience. It can provide insights into the fundamental principles of structural organization in the human brain and enhance our knowledge of how the brain processes and integrates information from various sources in real time (Sporns et al., 2004).

The structural foundations of the macroscopic level human connectome have been extensively studied using diffusion magnetic resonance (dMRI) scans (Bassett & Sporns, 2017; Fornito & Zalesky, 2013; Hagmann et al., 2008; Sporns et al., 2005; Zhang et al., 2018). This research field represents the human connectome as a graph with gray matter brain areas as nodes and axonal fibers as edges. This graph can be either binary or weighted. In binary graphs, edges only indicate whether a pair of nodes is directly connected or not. It commonly defined by the existence of axonal fibers connecting them, which can be delineated using dMRI-based fiber tractography, despite its inherent limitations (Maier-Hein et al., 2017). In weighted graphs, the edge values represent the strength of the connection and can be measured in different ways. The number of streamlines (NOS) connecting each pair of brain areas in the structural connectome (SC) is typically used to weight the connections. This weighted connectome has revealed several basic phenomena in the brain topology related to real-world networks such as small-worldness and scale-free behaviors (Bullmore et al., 2009; Gong et al., 2009; Hagmann et al., 2007, 2008). The main problem with counting the number of reconstructed streamlines as connection strength, is that such trajectories are an abstraction of the tractography algorithm itself and may not correspond to the actual number of axonal fibers, thus leading to potential inaccuracies in some cases (Calamante, 2019; Jones et al., 2013). Additionally, fiber tracking often lacks specificity due to various factors, including the choice of tractography algorithm and image acquisition parameters (Jones, 2010).

Researchers have also used different diffusion tensor imaging (DTI) indices to weigh links and investigate the structural connectome (Bastiani et al., 2012; Caeyenberghs et al., 2016; van den Heuvel & Sporns, 2011; Zhang et al., 2018). The underlying assumption is that variations in these measures reflect the integrity of the fiber tract and thereby affect the functional capacity of the connection between regions. For instance, fractional anisotropy (FA) provides insights into the microstructural properties of white matter (WM), but its value is influenced by various tissue properties, including axonal diameter, fiber density, tissue geometry, and degree of myelination (Basser & Pierpaoli, 2011; Jones et al., 2013).

Undoubtedly, comprehending the properties and functioning of the SC is of utmost importance in neuroscience. However, the definition of its connections remains a topic of debate. Although dMRI-based measures enable the estimation of connection strength, the most biologically informative weighted measure for representing anatomical connections is still uncertain (Fornito & Zalesky, 2013).

Recently, a novel method called axonal spectrum imaging (AxSI) has been proposed to estimate the axon diameter distribution (ADD) of streamlines in the human brain (Gast et al., 2023). This method utilizes a multishell dMRI framework to estimate the average ADD index for each reconstructed streamline. The AxSI framework builds upon a series of technological advancements in this domain (Assaf et al., 2004, 2008; Barazany et al., 2011; Veraart et al., 2020). Nevertheless, it showcases various significant benefits. Notably, its robust and simple modeling, which increases its applicability and make it compatible with a variety of scan protocols, including some existing scans like the HCP database (Gast et al., 2023). Since axon diameter is closely related to properties such as conduction speed, further development of such methods in conjunction with graph analysis tools could provide a physiologically meaningful framework for estimating the strength and conduction delay of anatomical connectivity between regions.

The ability to understand the properties of the SC is crucial in order to elucidate the infrastructure behind cognitive abilities, behavior, and traits. Various studies have previously demonstrated the ability of SC features to predict dysfunctions and diseases (Collin et al., 2015; Kuceyeski et al., 2016; Meier et al., 2020; Tymofiyeva et al., 2019), age (Zhao et al., 2015), and cognitive abilities (Feng et al., 2022; Seguin et al., 2020; Wiseman et al., 2017). However, as there are different ways of defining structural connectivity, it could affect the strength of the relationship between the SC and cognition, behavior, or psychopathology. Therefore, there is a need to quantify how such relationships are affected by different weighting approaches.

In this study we chose to compare the ADD-weighted SC, with the commonly studied NOS and FA SC. We characterized the different weighted SC measures, by presenting their weight, node degree, and nodal efficiency distributions, community partitions, and small-world structure. Our analysis was conducted on a group of 759 healthy young adults from the Human Connectome Project (HCP) dataset (Van Essen et al., 2013). We utilized an extreme gradient boosting model to predict total cognitive composite scores, which represent a general score of total intelligence (both crystallized and fluid abilities) assessed with the National Institutes of Health (NIH) Toolbox Cognition Battery (Weintraub et al., 2016) by each of the weighted SC measures. Intelligence score has been chosen for prediction analysis due to several previous studies that demonstrated its link to different properties of the human brain SC (Feng et al., 2022; Krupnik et al., 2021; Levakov et al., 2021; Popp et al., 2023).

## RESULTS

### Weighted Structural Connectomes

**Figure 1.**
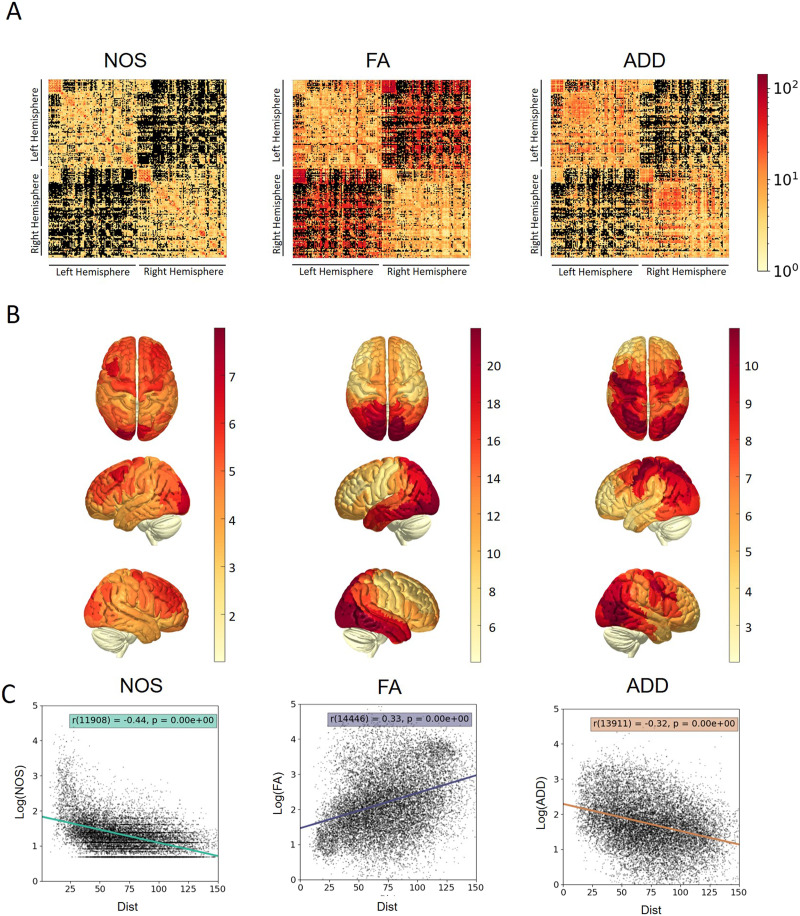
Weighted structural connectomes. (A) Connectivity matrix of average weights, weighted by the number of streamlines (NOS), by the mean FA index, and by the mean axon diameter distribution index (ADD). (B) Surfaces weighted by average weight. The figure shows the average weight value of all streamlines connected to each brain region from the atlas (Yeo 7-Networks), from three different points of view. Darker color (redder) represents higher values (stronger connections). (C) Edge-based Pearson’s *r* correlation and scatter plots to compare each histogram-matched weighted connectome (in logarithmic scale) with the Euclidean distance between brain regions centroids. Black dots represent edge samples and colored line for the resulted relation (linear regression fitting).

The connectome of each weighting method (NOS, FA, and ADD), after histogram matching, resulted in different connectivity matrices as demonstrated in Figure 1A. These matrices exhibited unique patterns of weight distribution. For example, the NOS SC exhibited the strongest connections between close brain areas, while the ADD SC displayed stronger links that were more widespread within each hemisphere. The FA SC, on the other hand, showed the strongest connections between the two hemispheres (commissural). Figure S1A showcases the weight distribution of all weighted SC, both before and after histogram matching normalization. This figure demonstrates the noticeable differences in distribution (shape and scale) before normalization, which are alleviated after histogram matching.

The distribution of the weighted edges in different ways becomes even more pronounced when looking at the surface representation of weights, as illustrated in Figure 1B. Each brain area is colored based on the average weight of the links connecting it with other areas. For instance, the ADD SC has most of its strong links connected to the parietal and occipital areas, while the FA SC shows strong connections with the occipital and temporal lobes, and the NOS SC exhibits evenly spread weights, with higher values in the frontal lobe. Comparing the average weights in different weighting methods resulted in insignificant correlations between most brain areas, but some areas showed positive or negative correlations that were spread out, as shown in Figure S1B. The overall correlation between edge weights resulted in positive correlation between ADD and NOS (*r* = 0.16, *p* << 0.01) and negative correlations for FA-NOS and FA-ADD (*r* = −0.14, *p* << 0.01 and *r* = −0.09, *p* << 0.01 accordingly). However, the trends do not stand out.

Figure 1C presents scatter plots and fitting of each weighted SC with the Euclidean distance between the corresponding centroids of brain areas (Dist). The results demonstrate positive correlation between logarithmic scale of FA and Dist (*r* = 0.33, *p* << 0.01) and negative correlation between logarithmic scales of NOS or ADD and Dist (*r* = −0.44, *p* << 0.01 and *r* = −0.32, *p* << 0.01, respectively).

### Node-Level Graph Properties

**Figure 2.**
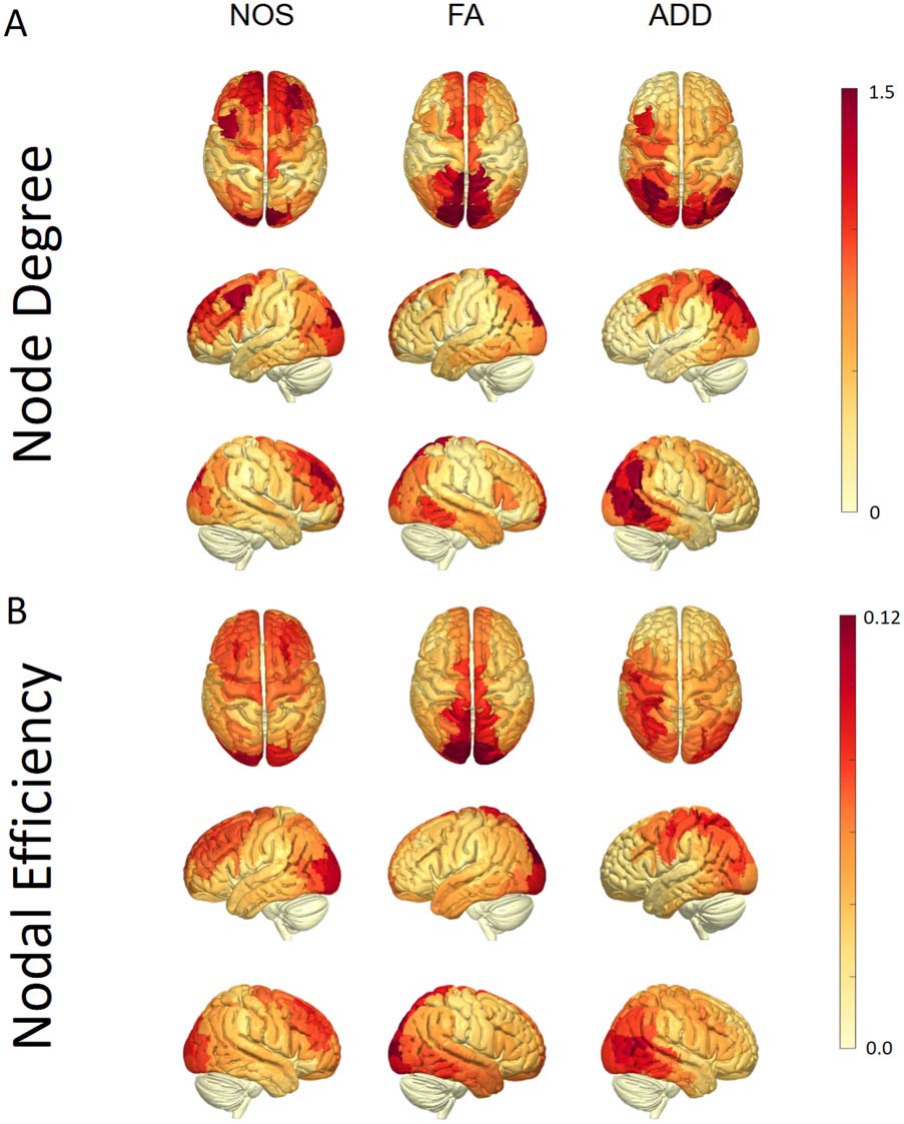
Node degree and nodal efficiency surface representation. (A) Surfaces are weighted by the node degree (for the entire HCP subjects’ group) in each brain area for NOS, FA, and ADD-weighted structural connectome and presented in three different directions. (B) Surfaces are weighted by the average nodal efficiency (for the entire HCP subjects’ group) in each brain area for Num, FA, and ADD-weighted structural connectome and presented in three different directions. Darker colors represent higher nodal efficiency (see color bars).

To further investigate the discrepancies between the weighted SC, we computed their node degree and nodal efficiency. The results, illustrated in Figure 2, display the averaged values across all subjects. Notably, the ADD SC exhibited asymmetry, with higher values in the node degree and nodal efficiency observed in the left hemisphere’s parietal lobe compared to the same areas in the right hemisphere. For the NOS SC, the highest node degree areas were primarily located in the frontal areas, while the highest nodal efficiency was situated both in the frontal areas and around the primary visual cortex. Lastly, the highest values for the FA SC were concentrated around the midline of both hemispheres.

### Community Detection

**Figure 3.**
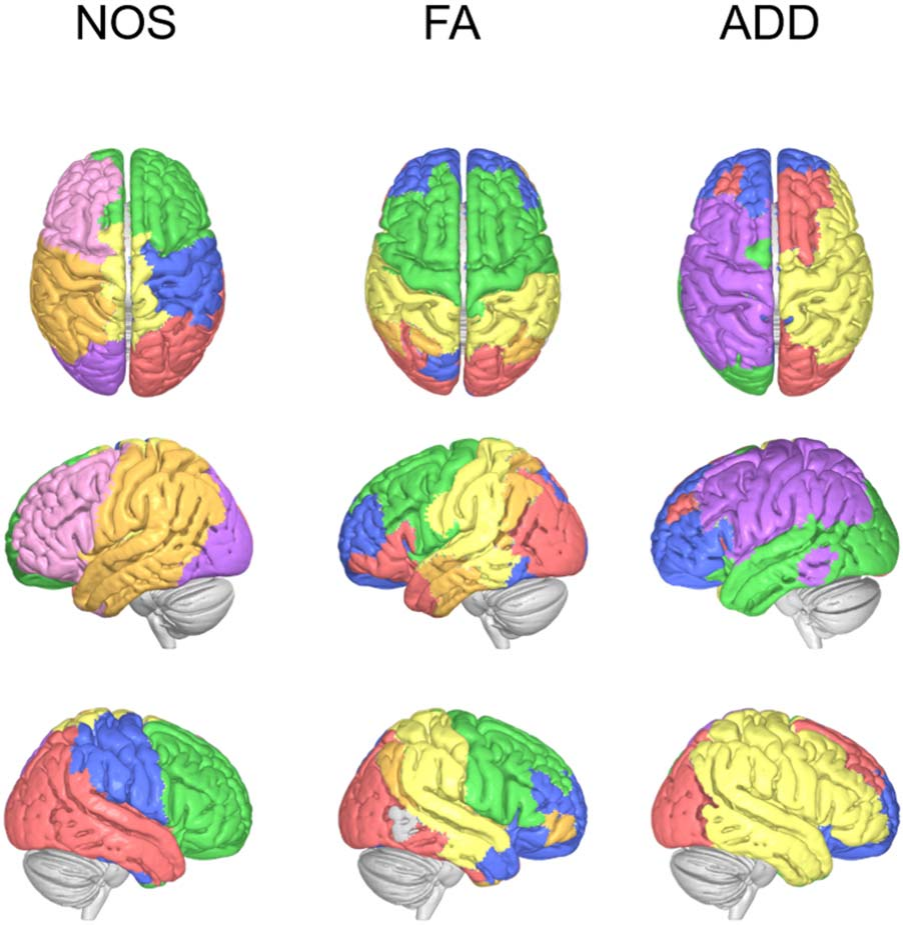
Louvain method for community partition results using the Yeo 7-networks atlas. Different colors in surface presentation represent different communities (separately in each weighting method).

Subsequently, we compared the different SC by examining their node partitioning based on link strength. We utilized the Louvain method for community detection on the connectivity matrices generated using the Yeo 7-networks atlas, which resulted in varying partitioning for the different weighted connectomes, as depicted in Figure 3. The NOS SC yielded seven different communities, while the FA and ADD SC produced only five. One notable difference was the mostly unilateral communities observed in the NOS and ADD-weighted SC, as opposed to the bilateral communities in the FA weighted SC. In other words, parallel areas in the left and right hemispheres were often related to the same community in the FA weighted SC. However, this was only true for some frontal areas in the ADD SC, whereas in the NOS SC, some medial areas from both hemispheres shared the same community. Similar results were obtained when applying community detection on connectivity matrices generated using the Briannatome atlas, as illustrated in Figure S2.

### Small-Worldness

**Figure 4.**
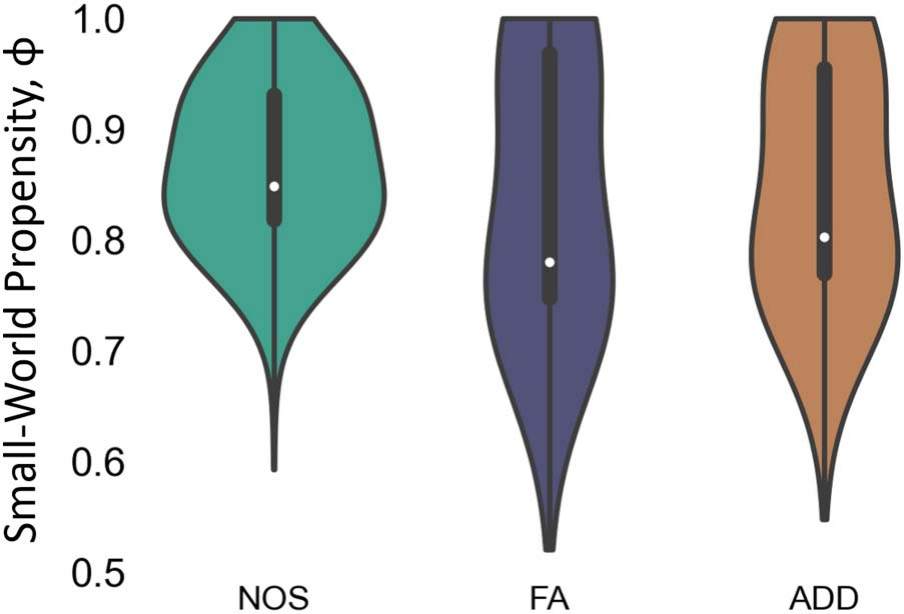
Small-world propensity values for each weighted SC. The violin plot represents the SWP median (white dot), quartiles (inner boxes) and kernel density (violin) of NOS (green), FA (purple), and ADD (orange) weighted SC.

The human brain SC has been shown to demonstrate small-world characteristics. We examined the small-world structure of the different weighted SC using small-world propensity (SWP) measure (Φ). Networks with high small-world characteristics, will have a value of Φ close to 1, while lower values of Φ indicate larger deviations from the respective null models for clustering and path length, and less small-world structure. Values of Φ greater than 0.6 are generally considered to exhibit a small-world structure. As shown in Figure 4, all three weighted connectomes displayed small-world structures, at different levels of small-worldness (*F* = 151, *p* << 0.001). The median values for SWP were Φ_NOS_ = 0.85, Φ_FA_ = 0.78, and Φ_ADD_ = 0.8. Post hoc *t* tests revealed that Φ_NOS_ had a higher value than Φ_FA_ (t_NOS-FA_ = 12.4, *p* << 0.001) and Φ_ADD_ (t_NOS-ADD_ = 12.3, *p* << 0.001). Furthermore, Φ_ADD_ had a higher value than Φ_FA_ (t_ADD-FA_ = 11.25, *p* << 0.001). All *p* values were corrected with Bonferroni correction for three comparisons.

### Prediction of Intelligence

**Figure 5.**
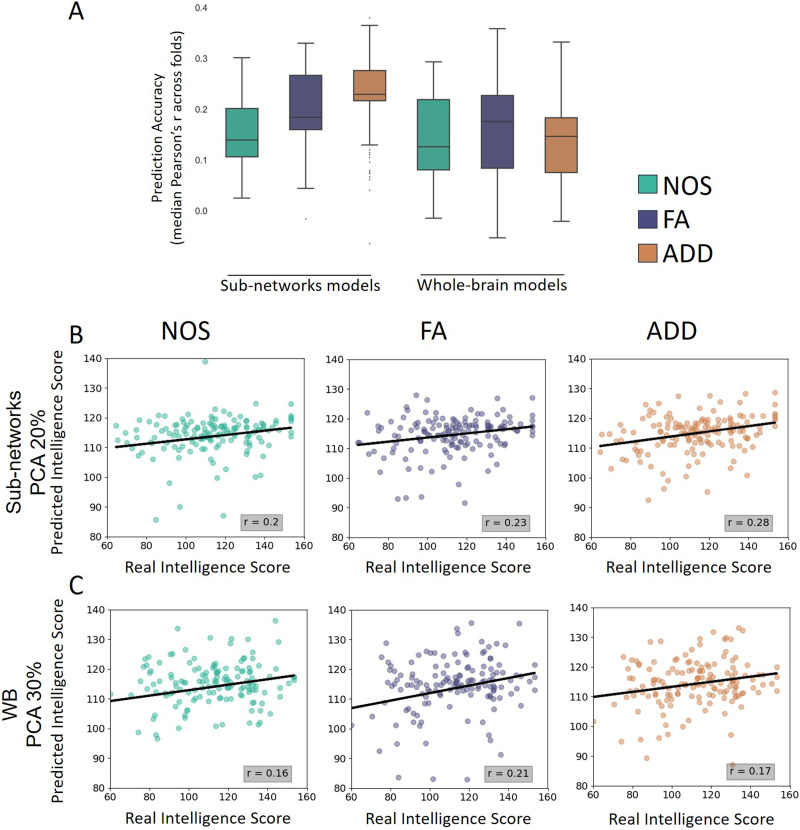
Total intelligence prediction using each weighted SC. (A) Accuracy results for subnetworks model with PCA components explain 20% of variance for each subnetwork and whole-brain (WB) model with PCA components explain 30% of variance total. Accuracy was measured as the mean Pearson’s *r* across folds. The box plot represents the median (solid line), quartiles (boxes) outlines (whiskers) and outliers (dots) of NOS (green), FA (purple), and ADD (orange) weighted SC for subnetworks models (left) and whole-brain models (right). Prediction accuracies were compared using Wilcoxon’s signed rank test and *p* values were corrected using Bonferroni correction for multiple comparisons, for 15 comparisons. (B) Scatter plot with values (circles) and their linear fit (solid line) for the model predicted values against the real values of test group subjects using PCA components that explains 20% of the variation of each subnetwork (subnetwork model, 23 subnetworks total, see Methods). (C) Scatter plot with values (circles) and their linear fit (solid line) for the model predicted values against the real values of test group subjects using PCA components that explains 30% of the variation of values from the entire brain (WB model). Pearson’s *r* values reported for each of the above-mentioned models.

Finally, we utilized different weighted SC to predict intelligence scores from the HCP dataset. Optimal hyperparameters for learning rate and number of boosting iterations were selected based on cross-validation analysis, with values of 0.05 and 200, respectively (Figure S3). Subnetwork models using FA and ADD SC performed significantly better than whole-brain models, as determined by Bonferroni-corrected *p* values for 15 comparisons (*p* < 0.0001; Figure 5A and Table S1). Among the subnetwork models, the ADD SC subnetworks model demonstrated the highest accuracy compared to any other model (*p* << 0.0001; Figure 5A and B and Table S1). Additionally, the FA SC subnetworks model performed significantly better than the NOS SC subnetworks model (*p* << 0.0001), while the FA whole-brain model performed significantly better than the NOS and ADD SC whole-brain models (*p* << 0.0001). Other models, including subnetworks models with principal component analysis (PCA) explaining 10/30% of variance for each subnetwork and whole-brain models with PCA explaining 20/40% of variance for the entire network, presented lower accuracies of prediction models (Figure S4). The number of predictors for each weighting method and each model, is presented in Table S2.

Most of the “partial” models, which excluded the component derived from different groups of subnetworks, did not result in a significant change in accuracy compared to the “full” model (with all components), except for the ADD SC subnetworks model, which had a significantly lower accuracy when no component from the left hemisphere was included (Figure S5 and Table S3).

## DISCUSSION

The network architecture of the human brain, known as the human connectome, is a subject of great interest. The human connectome is often represented as a graph with gray matter regions as nodes and axonal fibers that connect them as edges. This is referred to as the human brain connectome (Sporns et al., 2005). However, there is currently no consensus on the most effective method for assessing the strength of these connections, mainly due to continuous fail in demonstrating high functional or behavioral correlations, and hence to accept one weighting method as preferred. In this study, a new approach was presented that involves weighting connections based on their mean axon diameter using the AxSI method (Gast et al., 2023), in addition to commonly used methods such as the NOS reconstructed from fiber tracking analysis or the FA index from DTI method.

The results of this study reveal that the different methods used to weight connections provide distinct pictures of the structural network of the human brain. For a start, the distribution of connection strengths varies across methods (Figure 1), with the highest weights in the ADD-weighted SC being spread across both hemispheres and primarily connected to parietal and occipital areas, while the FA SC exhibits its highest weights between occipital and temporal lobes of both hemispheres. The strongest connections in the NOS SC are concentrated between close brain areas, particularly in frontal areas.

In brain networks, connection strength often decays with distance (Ercsey-Ravasz et al., 2013; Roberts et al., 2016; Rosen & Halgren, 2021), while FA shown to be positively correlate with edge length (Roberts et al., 2017). Figure 1C demonstrates a clear connection between all weighted SC (in logarithmic scale) to Euclidean distance. FA demonstrates a positive correlation, and NOS presents an opposite link, as previously shown. ADD, similar to NOS, presents a negative correlation with distance, meaning weaker connection for more distant brain areas.

These differences persist as more complex graph properties are examined, as demonstrated in Figure 2. For nodal properties, the ADD-weighted SC shows asymmetry in values, with higher node degree and nodal efficiency in areas of the left hemisphere. In contrast, the NOS SC demonstrates a dominance of the frontal lobe as a hub area of the brain network when measuring connection strength by counting the number of reconstructed streamlines. For the FA SC, the dominant areas are closer to the center of the brain structure. While different hypotheses might explain the ADD-weighted SC asymmetry, further work needs to be conducted to explore why this asymmetry exists. One possible explanation is left-hemisphere dominance for language functioning (Geschwind & Levitsky, 1968), which may correspond to more strongly connected brain areas, possibly in terms of axon diameter, in the left parieto-occipital regions. Although this explanation lacks prior evidence, a recent study found a link between the volume of the corpus callosum, left-sided intrahemispheric functional connectivity, and performance in different language domains among children (Bartha-Doering et al., 2021). This study suggests a possible link between WM microstructural variation and language performance.

When studying the SC, researchers must choose carefully which method to use for estimating links’ strength, since it affects network properties and, therefore, research conclusions. This decision is first and foremost affected by the research hypothesis. However, some consideration might affect this decision. The topology of NOS SC resembles more other real-world networks, with many lightly connected edges and few hubs (Bullmore et al., 2009; Gong et al., 2009; Hagmann et al., 2007, 2008). It is also easy to interpret more links as a stronger connection between brain areas. On the other hand, as discussed in the Introduction, the NOS suffers from high sensitivity to the tractography algorithm chosen and is therefore easily biased. Another consideration is the ability to biologically interpret the measure property of WM. In that case, ADD or myelin content might be a better candidate over FA or other general DTI indices for weighting edges. Being in correlation with axon conduction velocity (Drakesmith et al., 2019; Horowitz et al., 2015; Ritchie, 1982), ADD stands as a biologically relevant candidate to study brain function and efficiency as well.

Several studies examined the effect of different decisions during the process of connectomes creation on the resulted connectomes (Buchanan et al., 2020; Gajwani et al., 2023; Oldham et al., 2020). Recent work by Gajwani et al. (2023) has shown that the choice of dMRI data processing strategy influences how important nodes are identified. Their analysis, based on different NOS connectomes (both binary or weighted) shows that each step in the process, from choosing an atlas, the tractography method used through the thresholding, all might affect node importance distribution. A different study conducted on data from the UK Biobank demonstrated an effect of thresholding of edge weights on age-sensitive measures resulted from probabilistic tractography-weighted connectomes (Buchanan et al., 2020). Both studies correspond with the results presented here, where all parameters were kept constant apart from the choice of weighting method, which resulted in varied nodes importance between the different methods. This result emphasizes how selecting different processing steps can shape the SC, and more broadly how different weighting methods can affect tractography derived results.

In addition to the differences in nodal properties, the global structure of the human brain connectome was also examined using the different weighting approaches. The results showed that there were differences in community partitioning between the three methods (FA, ADD, and NOS). The FA method resulted in mostly bilateral communities, while the ADD and NOS methods showed mostly unilateral communities. However, the number and grouping of the communities differed between the two methods (Figure 3). It was also found that all three methods exhibited a small-world structure, but the NOS SC had the highest level of small-worldness in the network (Figure 4).

The study’s analysis of the different weighted networks did not reveal a preferred approach, but rather highlighted the unique information provided by each method. However, when the networks were used to predict intelligence scores from the HCP dataset, the subnetworks of the ADD-weighted SC showed the strongest predictive power (Figure 5). The ADD index, which is an estimation of the average axon diameter of the streamlines connecting pairs of nodes, is known to be correlated with axon conduction velocity (Ritchie, 1982; Waxman, 1980), a meaningful microphysiological property of white matter tissue that may be more relevant for estimating cognitive abilities.

The study also found that using the PCA compartments derived from different subnetworks separately was more successful in prediction models than using compartments from the entire brain together (WB models). This suggests a link between the SC and behavior that is mediated through the brain’s functional domains. Future research on other behavioral modalities may provide further insights into the relationship between the functional connectome and the underlying structural network.

The study aimed to explore the differences in the human brain connectome’s structural network when using various methods to weigh the strength of its links. The results showed that each of the methods produced distinct characterizations of the network. However, since the connectome is inherently directed, representing it with undirected edges may limit the accuracy and interpretation of its graph-based representation.

Furthermore, our study highlights that there is no one preferred approach for weighting the strength of graph edges in the human brain network. While the ADD-weighted SC showed better performance in predicting intelligence, it is possible that other cognitive domains or behavioral traits may be better captured by other weighting methods. In order to determine which weighting method best captures the relationship between brain network structure and function, a thorough investigation of the functional connectome representation to the underlying structure is necessary. One possible direction to further study these relations might be to study the resemblance between each weighting connectomes to the related fMRI-based functional connectome. Previous studies have attempted to explore this structural-functional relationship by weighting SC edges with reconstructed streamlines or a combination of structural properties (Abdelnour et al., 2014; Amico & Goñi, 2018; Meier et al., 2016; Park & Friston, 2013; Rosenthal et al., 2018). However, there is still much to be discovered in this field, and finding the most appropriate weighting method remains a challenge. One possible new approach might be to find a combination (adding, multiplying, or other) of structural properties to define the structural connections strength.

Recently, a study by Nelson et al. (2023) examined different weighted connectomes, weighted by different properties of the WM. Their study showed that SC weighted by myelin (R1) and edge caliber (measure using COMMIT) were most related to FC. Although using a different method to estimate axon diameter, their results are in line with the results here, where the ADD-weighted connectome was most successful in predicting cognitive abilities. Both studies emphasize the need of an index for WM microstructure to weight the SC in order to be able to interpret its relation with function and behavior. While their work systematically compared different approaches with FC and distance measures, here we supplement the ability of the weighted SC to predict cognitive abilities that previously seldom resulted in poor ability if any.

The significance of understanding the SC and its relation to brain functions cannot be overstated. Properly defining the connections’ strength is a crucial aspect of this endeavor. Most studies thus far have focused on diffusion MRI measures and streamline reconstruction. Our study presents a novel microphysiology relevance approach based on the AxSI method, which estimates the axonal diameter throughout the brain. Given its correlation with conduction velocity, it is reasonable to assume that it has relevance in estimating connection strength and studying graph properties in relation to function.

## METHODS

### Data

Seven hundred and fifty-nine randomly selected healthy adults (397 females, age 22–37 years, mean 28.7 years) from HCP 1,200 young adults release (Van Essen et al., 2013) were used in this study. The decision to use a subset of subjects from the entire dataset was made due to processing time considerations.

### Scans

Subjects were scanned on a 3T Magnetom Siemens Skyra scanner (Siemens, Erlangen, Germany) with a 128-channel radiofrequency coil and customized SC72 gradient system reaching 80 mT/m. In this study the following sequences were used:

1. A multishell diffusion-weighted imaging sequence, with Δ/*δ* = 43.1/10.6 (ms) and b-shells of 1,000, 2,000, and 3,000 (s/mm^2^).
2. An MPRAGE sequence, with TR/TE = 2,400/2.14 (ms).

Full protocol details available in the HCP reference manual (https://www.humanconnectome.org/storage/app/media/documentation/s1200/HCP_S1200_Release_Reference_Manual.pdf). Subject recruitment procedures and informed consent forms, including consent to share deidentified data, were approved by the Washington University institutional review board.

### Intelligence Scores

The intelligence scores were obtained from the Human Connectome Project (HCP) dataset, using the total composite score from the NIH Toolbox Cognitive Function Battery (Weintraub et al., 2016). The battery consists of seven tests that assess cognitive abilities essential for adaptive functioning across the life-span, including Picture Vocabulary, Reading Tests, Flanker, Dimensional Change Card Sort, Picture Sequence Memory, List Sorting, and Pattern Comparison. The composite score was calculated by averaging the normalized scores of each test and deriving scale scores based on this new distribution. Higher scores indicate higher levels of cognitive functioning. Participant scores were normed using the age-appropriate band of the Toolbox Norming Sample, which included bands of ages 18–29 or 30–35. A score of 100 indicates performance at the national average, while scores of 115 or 85 indicate performance one standard deviation above or below the national average for the participant’s age band.

### Preprocessing

The HCP provides minimally preprocessed images (Glasser et al., 2013). This preprocessing pipeline includes intensity normalization across runs, *topup* and *eddy* corrections, gradient nonlinear correction, and registration of MPRAGE to diffusion image using the Functional MRI of the Brain (*FMRIB*) linear image registration tool boundary-based registration (*BRB*). The full pipeline is available online (https://github.com/Washington-University/Pipelines).

### Tractography

The fiber tracking analysis on diffusion scans was conducted using the Mrtrix3 software package (Tournier et al., 2019), which uses an MSMT- CSD reconstruction (Dhollander et al., 2016; Jeurissen et al., 2014; Tournier et al., 2019), followed by anatomically constrained deterministic tractography. Analysis was done using the SD_stream algorithm in MRtrix3, extracting four million streamlines per subject. To extract the full tractogram, we used the entire brain mask as a seeding mask and then filtered the tracts using the MRtrix3 anatomically constrained SIFT algorithm (Smith et al., 2013) so that 40,000 tracts remained extracted for each tractogram.

### Weighted Structural Connectomes

Connectivity matrices were created using either Yeo-7 networks (Schaefer et al., 2018) with 200 areas (100 nodes for each hemisphere) or Brainnetome (BNA) atlas (Fan et al., 2016) with 274 areas (123 nodes for each hemisphere and 28 cerebellar nodes), as defined nodes. These atlases are divided to provide functional connectivity segmentation of the cortex, allowing a base to explore relations between weighted SC and function. In each case, each pixel in the matrix represents the edge weight of links between a pair of brain areas (nodes), located in the relevant row and column. The weights were calculated as follows:

- NOS Structural Connectome: the number of streamlines that connect the pair of brain areas, as reconstructed from tractography (see previous section).
- FA SC: each reconstructed streamline has been weighted by the average FA of all the voxels it passes through. The weight was calculated as the mean FA value for all reconstructed streamlines that connect each pair of brain areas.
- ADD SC: Using AxSI analysis (Gast et al., 2023), the average ADD per voxel was calculated. Then, estimated mean axon diameter (eMAD) per streamline was calculated. Finally, the weight was calculated as the mean ADD value for all reconstructed streamlines that connect each pair of brain areas. To be precise, the volume used as weights for weighted connectome is the eMAD. However, to avoid confusion between average along streamline or between streamlines that connects each pair of nodes or between subjects, the ADD is used as the weighting name and noted when it is averaged.
- Dist SC: For the analysis of relation between edge weight and nodes distance, a distance matrix was calculated as the Euclidean distance between the centroids of each pair of nodes.

### AxSI Analysis

AxSI is a framework for estimating axonal diameter distribution per extracted streamline, based on high angular resolution diffusion imaging (HARDI) scans (Gast et al., 2023). It models the measured signal as the linear combination of three water pools:

1. Cerebral spinal fluid (CSF), where water molecules diffuse freely, described as gaussian distribution
2. Cellular and extracellular hindered diffusion, described as diffusion tensor
3. Axonal restricted diffusion, described as motion within impermeable cylinder

The ADD estimation is done using a linear fit of the measured signal for a series of 160 predetermined axon diameters that cover the range of possible diameters in the CNS. This MRI signal library, which also includes gaussian and tensor distributions (for CSF and Hindered, accordingly), is fitted to the measured signal. Moreover, a regularization is applied to ensure fitting optimization and avoid overfitting and noisy distribution of diameters. This process is done voxel-based and applied on tractography to calculated the eMAD, which is done by averaging the relative contribution of each compartment and along the streamline. Finally, weighted ADD CM is calculated as described above. For more details regarding AxSI framework, see Gast et al. (2023).

### Histogram Matching

Due to the distinct weight distribution shape and range of values in each of the above-mentioned methods for weighting links in the SC (refer to Figure S1A in the Supporting Information), it is challenging to compare between them. To address this issue, weighted SC were histogram-matched for all network property analyses (Figures 1–3 and Supporting Information Figures S1 and S2).

For each subject, their structural connectome was adjusted to match the weight distribution of their NOS SC, and any values lower than 1 were excluded. The “match_histogram” method in the “scikit-image” package (van der Walt et al., 2014) was used for histogram matching, which aligns the cumulative histogram of one matrix to another. For small-worldness comparisons and intelligence prediction models, original matrices were utilized.

### Surface Representations

Network matrices weighted by the average weight of each SC were calculated as described above, including histogram matching. We then calculated the mean weight of each edge over the entire group of subjects (excluding zeros from calculation) to create a weighted network matrix of the group. We used median absolute deviation outlier detection for each edge, in order to exclude extreme values (Leys et al., 2013). These group matrices were used to calculate the average weight connecting each node in the average weighted network.

In the surface representation of the mean-weighted SC of brain areas, each area value is a representation of the mean weight value, presented on atlas surface representation using MATLAB *isosurface* function.

Node degree was calculated as the sum of weights for each subject separately and then averaged for the entire group for the surface representation. In which, each brain area is colored according to the average node degree for all subjects.

Nodal efficiency was calculated for each subject as the reverse of the mean shortest path from each node to every other node in the network. For this analysis calculation, matrices’ weights were reversed (so that stronger connection between nodes will represent shorter distance). After calculating the nodal efficiency for each subject, the values were averaged for each node (in each weighted SC) for the entire group to present over surfaces, in which, each brain area is colored according to the average nodal efficiency for all subjects.

Nodal weights correlations were calculated using Pearson’s *r* for each pair of weights. All *p* values were corrected using false discovery rate correction, and only significant *r* values were kept for presentation over brain surfaces.

### Community Detection

Community detection analysis was done using the averaged group matrices (described in the previous section). Community detection was done using the Brain Connectivity Toolbox (Rubinov & Sporns, 2010, 2011) and was based on the Louvain method for community detection (Blondel et al., 2008). For each weighted SC, the following steps were performed: first, we searched the relevant resolution parameter range (gamma) to use in the analysis. Included value consisted of values that returned more than three communities per network and less than N/4 (N - the number of nodes in the atlas) communities. Using this range limits, we use the same sampling rate of 1,000 samples (with weight specific range), across all weighted SC. We used a “consensus clustering” (Lancichinetti & Fortunato, 2012) approach, to sample many modular partitions across a range of gamma (in exponential sampling) to capture numbers of modules between 4 and N/4. Afterward, 1,000 modular partitions were aggregated into a coclassification matrix (using *agreement* function), and a consensus cluster was chosen using *consensus_und* function and a threshold (Tau) for weak elements of 0.2. Finally, a surface representation of communities was created similarly to the description in the previous section.

### Small-Worldness

Small-worldness was measured on each original weighted SC (before histogram matching) using SWP value, Φ, calculated as described in Muldoon et al. (2016), and using their MATLAB code for calculation. To quantify the extent to which a network display small-world structure, the SWP definition reflect the deviation of a network clustering coefficient, and characteristic path length, from lattice and random networks, constructed with the same number of nodes and the same degree distribution. Networks with high small-world characteristics, will have a value of Φ close to 1, while lower values of Φ represent larger deviations from the respective null models for clustering and path length, and display less small-world structure. Values of SWP > 0.6 are considered to have small-world structure (Bassett & Bullmore, 2017; Muldoon et al., 2016).

A repeated-measures ANOVA was done to compare the small-worldness of different weighted SC. Post hoc analysis consisted of pairwise *t* tests for each pair of weights (total of three possible pairs). All *p* values were corrected using Bonferroni correction for multiple comparisons, for three comparisons.

### Intelligence Prediction Models

Models to predict total intelligence score, as measured using the NIH Toolbox (see previous section) was created using whole-brain input or subnetworks input.

### Models Input Data

For all models, PCA was used to decrease the number of predictors in the model from all the links in the network to a subset of values represent their variation. Values of original weighted SC, in all models, were normalized and standardized for each subject, prior to the PCA analysis.

For each whole-brain model (using each weighted SC separately), input consisted of the minimal component from principal component analysis (PCA), needed to describe 20, 30, or 40% of the network data variation between subjects.

For each subnetworks model, connectivity matrices were first divided into 23 subnetworks; each subnetwork from the Yeo-7 functional networks atlas subdivision (visual, somatomotor, dorsal attention, ventral attention, limbic, fronto-parietal, default) was divided to connection inside each hemisphere (left\right) and connections inside the network but connecting the two hemispheres. For example, the visual network links were divided into links inside the left hemisphere, links inside the right hemisphere and links between hemisphere (of nodes related to the visual network). This sums up to a total of 21 subnetworks (three from each original network). Another two subnetworks were for connection inside each hemisphere, connecting different networks from the Yeo-7 networks definition. This subdivision provides a sense of functional-related subdivision to input variables of the prediction model. Then, for each subnetwork, model input consisted of the minimal component from PCA, needed to describe 10, 20, or 30% of the subnetwork data variation between subjects. Since the number of nodes in each functional network is different, and therefore the amount of data in each subnetwork is different, the number of PCA components to describe at least a certain percent of the data variation, varied between different subnetworks.

### Gradient Boosting Model

Gradient Boosting Algorithm, using XGBRegressor from XGBoost python-based library (Chen & Guestrin, 2016), was used to predict intelligence from the weighted SC components resulting from PCA. The number of trees for estimation (*n*) and learning rate (eta) were determined with tuning hyperparameters cross-validation process (see below). In each step, the algorithm learns the error received in a previous step of linear regression by using gradient descent to minimize squared error loss function. An L1 regularization (alpha = 1), which results in smoother final weights of the model, was applied to reduce overfitting.

### Tuning Hyperparameters

In order to decide which *n* and eta values to use, we applied a 10-fold cross-validation analysis, with the *GridSearchCV* class from scikit-learn library (Pedregosa et al., 2011). Models were evaluated using negative Mean absolute error (MAE) for all combinations of *n* = [10, 50, 100, 200, 300, 500] and eta = [0.005, 0.01, 0.05, 0.1]. Parameters were chosen from the plateau part of the graph (where MAE is minimal and stops improving).

### Assessment of Significant Differences in Prediction Results

In order to assess whether there is a significant difference between the accuracy of the predictions based on two different models (whole-brain\subnetworks for Num, FA, and ADD), we performed 1,000 iterations where we split the data randomly to training and test sets (4/5 train, 1/5 test), and predicted behavioral scores. In every iteration, prediction success for each dataset was measured by Pearson’s *r* between actual and predicted scores. Significant differences in prediction success were detected using a nonparametric, related samples test (Wilcoxon signed ranks test), which takes into account the overlap in training sets across iterations (Demšar, 2006).

We compared prediction success of six models, in a total of 15 comparisons. Therefore, all *p* values were corrected using Bonferroni correction for multiple comparisons, for 15 comparisons.

### Subnetwork Importance

We perform an analysis of subnetwork effects on prediction ability, for a subnetwork model with PCA that explains 20% of the variance in each subnetwork. In this analysis we iterate over the groups of subnetworks; seven functional networks from Yeo-7 parcellation, internetworks (links that connect nodes from different functional networks) and all the links in the left\right hemisphere (10 groups total) to create prediction model with all components except from the current group. For each group, we performed 100 iterations where we split the data randomly to training and test sets (4/5 train, 1/5 test), and predicted behavioral scores. In every iteration, prediction success for each dataset was measured by Pearson’s *r* between actual and predicted scores. Significant differences in prediction success against “full” model (which consist of all the subnetworks of the model), were detected using a nonparametric, related samples test (Wilcoxon signed ranks test).

We compared the prediction success of 10 models for each weight SC, in a total of 30 comparisons. Therefore, all *p* values were corrected using Bonferroni correction for multiple comparisons, for 30 comparisons.

## SUPPORTING INFORMATION

Supporting information for this article is available at https://doi.org/10.1162/netn_a_00342.

## AUTHOR CONTRIBUTIONS

Hila Gast: Conceptualization; Formal analysis; Investigation; Methodology; Software; Validation; Visualization; Writing – original draft. Yaniv Assaf: Conceptualization; Funding acquisition; Methodology; Supervision; Writing – review & editing.

## FUNDING INFORMATION

Yaniv Assaf, Israel Science Foundation (https://dx.doi.org/10.13039/501100003977), Award ID: 1303/20. Yaniv Assaf, United States - Israel Binational Science Foundation (https://dx.doi.org/10.13039/100006221), Award ID: 2018711. Yaniv Assaf, BIRAX 43BX18HJYA, ERC Grant number: 101054909.

## TECHNICAL TERMS

Graph: A mathematical description of a network comprising a set of nodes and a set of edges representing the pairwise relations between nodes.
Structural connectome: A network representation of the physical connections in the brain. Nodes represent brain regions, whereas edges represent physical connections of pairs of brain regions through the white matter.
Tractography: Computational reconstruction procedure that may be used to obtain, from diffusion MRI data, the white matter streamlines or fiber tracts connecting different brain regions.
Diffusion tensor imaging (DTI): A magnetic resonance imaging technique that measures the diffusion of water in tissue in order to produce axonal fiber tract images.
Fractional anisotropy (FA): A scalar DTI index to estimate how restricted and oriented the diffusion of water molecules is within a voxel or larger image volume.
Axonal spectrum imaging (AxSI): A diffusion MRI based method to estimate the axon diameter distribution.
Histogram matching: The process of transforming one distribution to match the distribution of the other.
Principal component analysis (PCA): A statistical procedure that uses an orthogonal transformation to convert a set of observations of possibly correlated variables into a set of values of linearly uncorrelated variables called principal components.
Prediction model: A mathematical process used to predict values by analyzing patterns in a given set of input data.
Diffusion MRI: MRI sequence quantifying the orientation of water molecule diffusion.
