# Supplementary material for: Weighting the structural connectome: Exploring its impact on network properties and predicting cognitive performance in the human brain
Source: Netw Neurosci. 2024 Apr 1;8(1):119–37. doi: 10.1162/netn_a_00342 (PMC10861171; doi:10.1162/netn_a_00342)
Supplement: Supplementary file 1 [file netn-8-1-119-s001.pdf]

Title:

Weighting the Structural Connectome: Exploring its Impact on Network Properties and Predicting Cognitive Performance in the Human Brain

Authors:

Hila Gast*, Yaniv Assaf

Included metrics:

Figs S1-5

Table S1-3

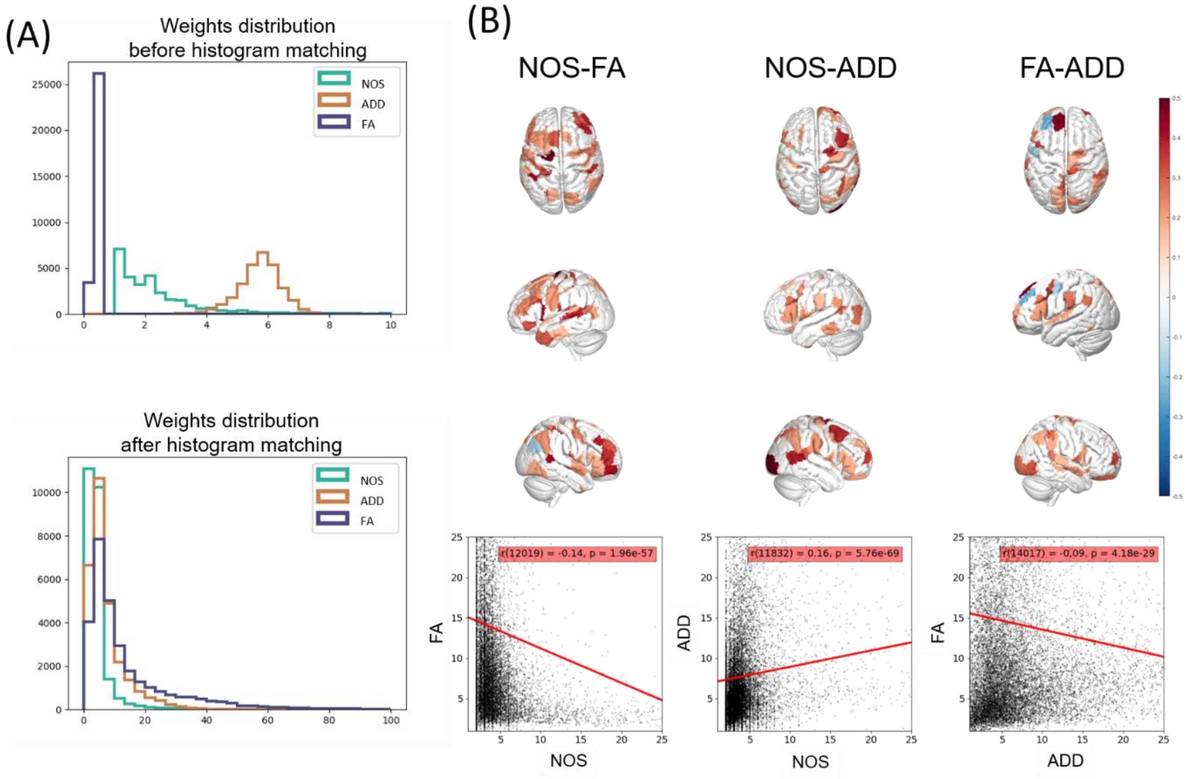

FigS1. (A) Weights distribution before and after histogram matching. (B) Pearson’s r correlations between node’s weights of NOS & FA (left), NOS & ADD (middle) and FA & ADD (right) connectomes. All p values were corrected using FDR correction for multiple comparisons and only significant r values presented. Positive correlation marked with red color schemes and negative correlations marked with blue color scheme (see colorbar). Lower row shows the edge-level Pearson’s r correlation and scatter plots for the same pairs.

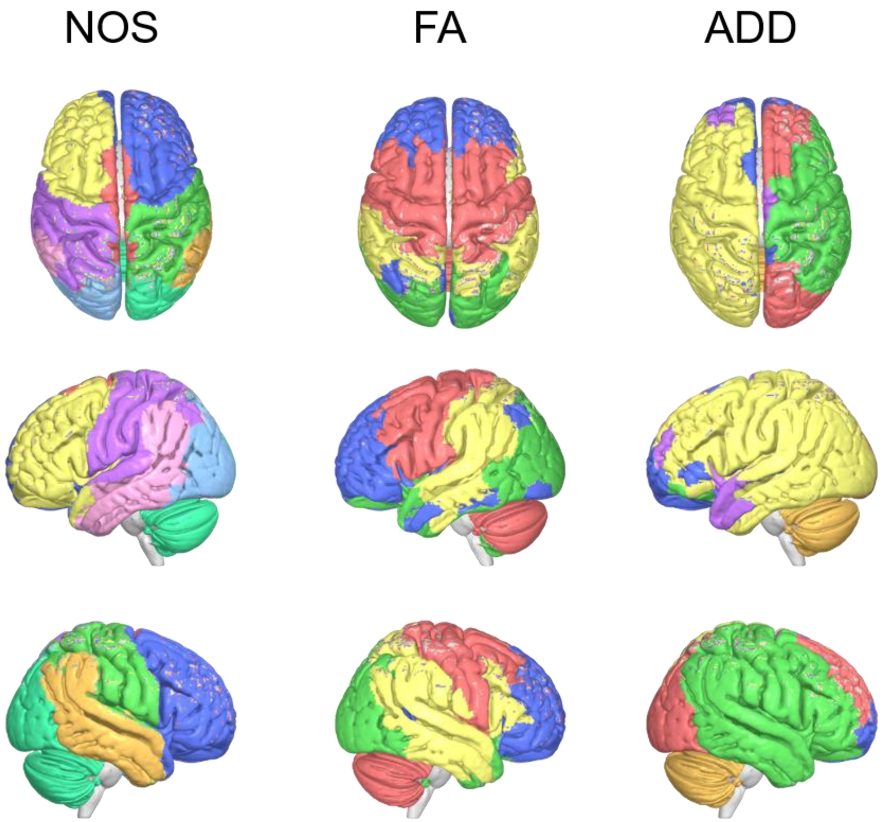

FigS2. Louvain method for community partition results using the BNA atlas. Different colors in surface presentation, represents different communities (colors are not matched between the methods and chosen arbitrarily).

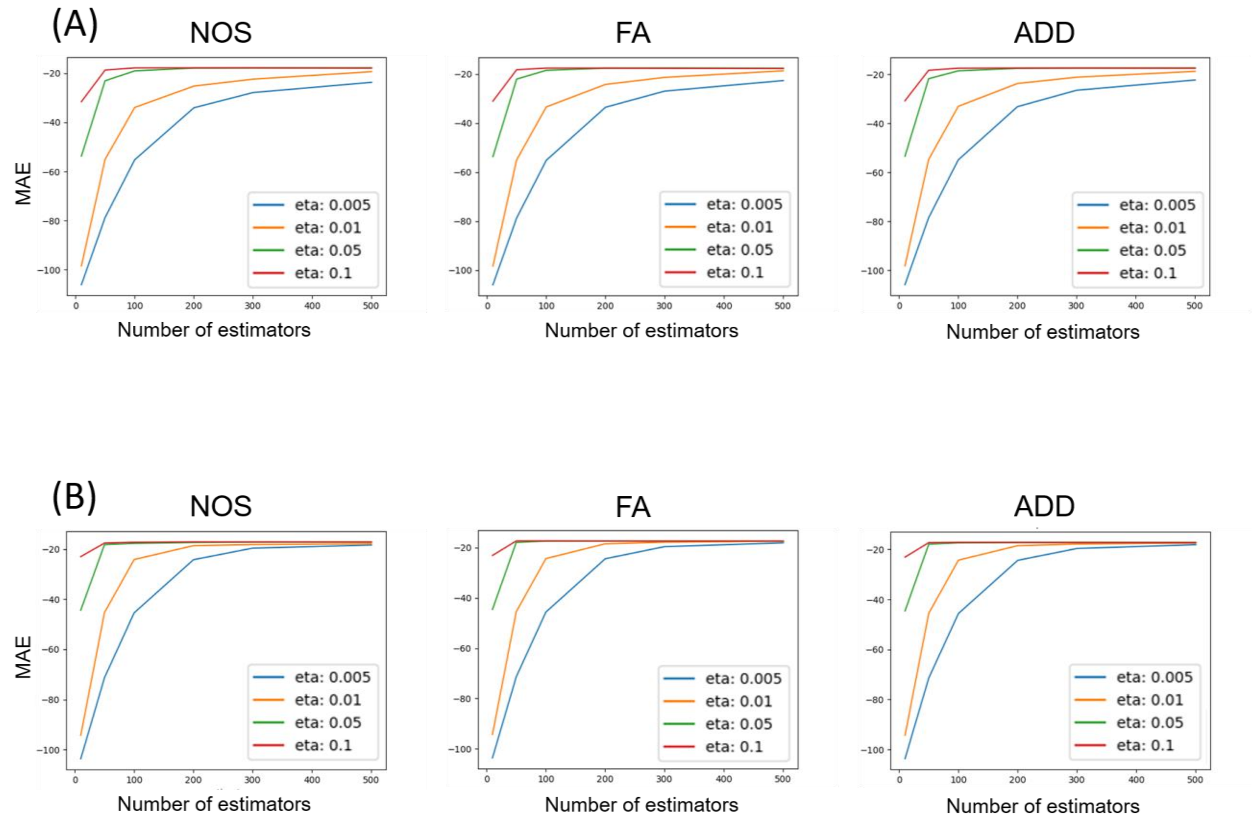

FigS3. Parameters tuning for extreme gradient boosting prediction models. Plots present the negative MAE values for sub-networks models (A) and whole-brain models (B) for different learning rates (0.005, 0.01, 0.05, 0.1) as different color lines (see legend) and different number of estimators (10, 50, 100, 200, 300, 500) as points on the graphs.

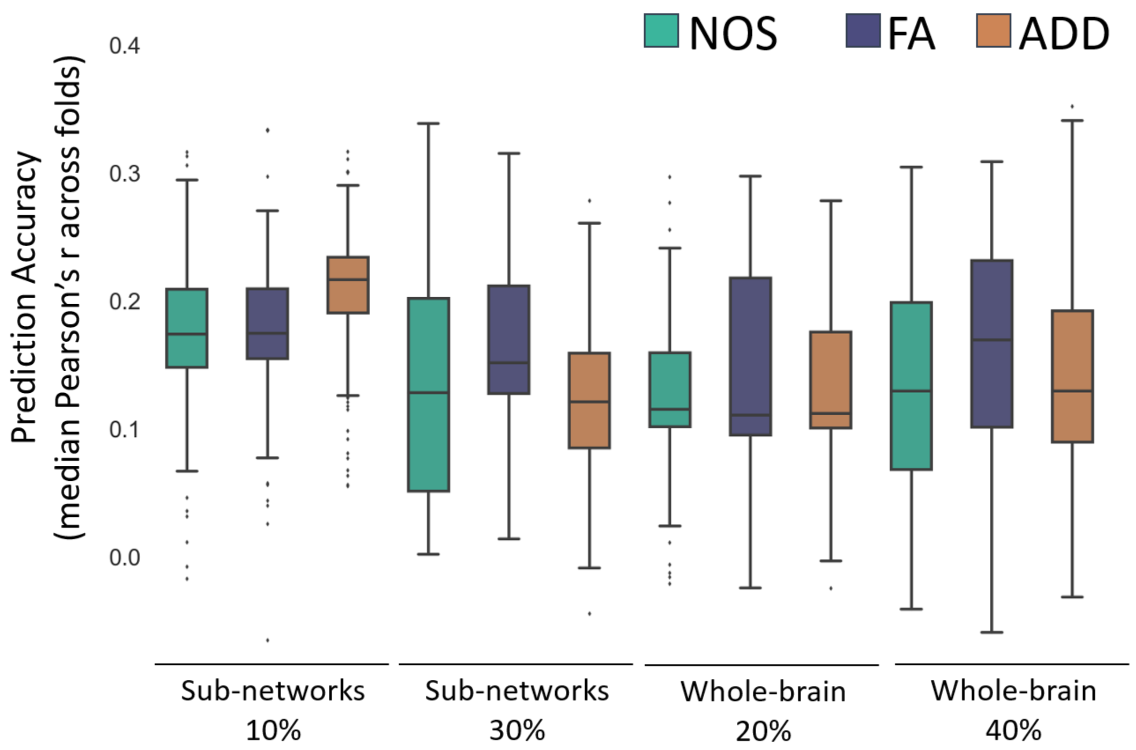

FigS4. Total intelligence prediction using each weighted SC. Accuracy results for more tested models; sub-networks with PCA explains 10% and 30% of each sub-network's variation and WB with PCA components explains 20% and 40% of variation total. Accuracy was measured as the mean Pearson's r across folds. The box-plot represents the median (solid line), quartiles (boxes) outlines (whiskers) and outliers (dots) of NOS (green), FA (purple) and ADD (orange) weighted SC for sub-networks models (left) and whole-brain models (right).

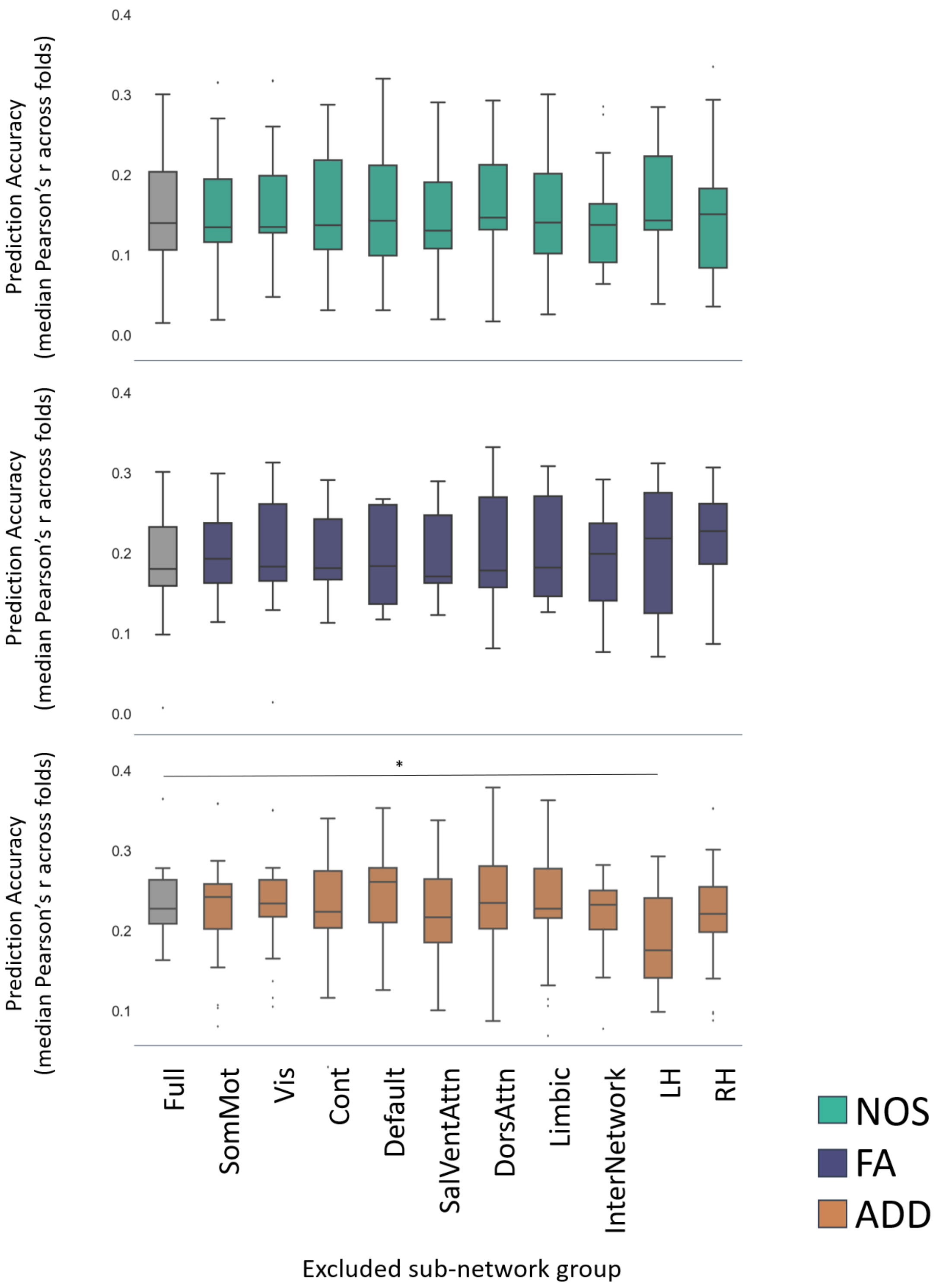

FigS5. Sub-network importance. Graphs show the model accuracy created using all of network components except the links of the components mentioned in x-axis. At the left side of each weighting method's graph, is the accuracy of the original model with all components included. See Sub-network important section in the methods for further details regarding the analysis.

Model 1	Model 2	W	p	p-corrected
NOS Sub-networks	FA Sub-networks	110312	5.80E-53	8.70E-52
NOS Sub-networks	ADD Sub-networks	37969	1.93E-119	2.90E-118
NOS Sub-networks	NOS WB	155557	3.57E-25	5.35E-24
NOS Sub-networks	FA WB	143822	2.30E-31	3.45E-30
NOS Sub-networks	ADD WB	100070	1.00E-60	1.51E-59
FA Sub-networks	ADD Sub-networks	110598	9.39E-53	1.41E-51
FA Sub-networks	NOS WB	68842	9.52E-88	1.43E-86
FA Sub-networks	FA WB	45819	6.52E-111	9.78E-110
FA Sub-networks	ADD WB	19695	1.57E-140	2.35E-139
ADD Sub-networks	NOS WB	23590	6.86E-136	1.03E-134
ADD Sub-networks	FA WB	12983	1.03E-148	1.55E-147
ADD Sub-networks	ADD WB	3698	2.03E-160	3.05E-159
NOS WB	FA WB	236814	0.141362	2.12E+00
NOS WB	ADD WB	216561	0.000226	3.39E-03
FA WB	ADD WB	225208	0.006122	9.18E-02

Table S1 Wilcoxon’s test results comparing model accuracies of sub-networks models with PCA explains 20% of variance for each sub-network and WB models with PCA explains 30% of variance total for all three weighted SC (NOS, FA, ADD). All p values corrected using Bonferroni correction for multiple comparisons, for 15 comparisons.

	**Sub-networks**			**WB**		
**% Explained variance**	**10%**	**20%**	**30%**	**20%**	**30%**	**40%**
**NOS**	82	200	355	28	50	78
**FA**	78	208	390	32	60	98
**ADD**	76	201	377	32	60	97

Table S2. The number of components in each model. Table presents the number of input component enter to each of WB or Sub-networks model to explain the percent of variance as listed in second row.

Weighted SC	Removed sub-networks group	W	p	p-corrected
NOS	SomMot	2500	0.931	1
	Vis	2053	0.104	1
	Cont	2490	0.904	1
	Default	2368	0.5893	1
	SalVentAttn	2260	0.36221	1
	DorsAttn	2132	0.176	1
	Limbic	2525	1	1
	InterNetwork	1985	0.063	1
	LH	2428	0.7387	1
	RH	2223	0.299	1
FA	SomMot	2250	0.344	1
	Vis	1958	0.051	1
	Cont	2450	0.796	1
	Default	2378	0.613	1
	SalVentAttn	2403	0.674	1
	DorsAttn	2163	0.213	1
	Limbic	2469	0.847	1
	InterNetwork	2456	0.8124	1
	LH	2506	0.9479	1
	RH	1719	0.00558	0.1674
ADD	SomMot	2396	0.657	1
	Vis	1996	0.0689	1
	Cont	2502	0.936	1
	Default	1631	0.0021	0.063
	SalVentAttn	2154	0.202	1
	DorsAttn	2015	0.0795	1
	Limbic	1946	0.0465	1
	InterNetwork	2311	0.4618	1
	LH	714	4.76E-10	1.42812E-08
	RH	2467	0.8419	1

Table S3 Sub-networks importance test results. Wilcoxon's test results compare models with removed groups of sub-network with a full model containing all sub-networks. All p values corrected using Bonferroni correction for multiple comparisons, for 30 comparisons.
